# Community-built environment and self-rated health in Western China: a latent serial mediation within SEM of sleep quality and family functioning

**DOI:** 10.3389/fpubh.2026.1787068

**Published:** 2026-04-24

**Authors:** Lansicheng Yao, Jacquline Tham, Qing Pan, Xiaobing Tian

**Affiliations:** 1 Foreign Affairs Office, North Sichuan Medical College, Nanchong, Sichuan, China; 2 Graduate School of Management, Postgraduate Center, Management and Science University, Shah Alam, Selangor, Malaysia; 3 Health Management Center, General Practice Medical Center, Innovation Institute for Integration of Medicine and Engineering, West China Hospital, Sichuan University, Chengdu, China; 4 School of Public Health, North Sichuan Medical College, Nanchong, Sichuan, China

**Keywords:** community-built environment, family functioning, self-rated health, sleep quality, structural equation model

## Abstract

**Objectives:**

To clarify the underlying relationship between community-built environment and self-rated health in rapidly urbanizing China and examine the chained mediation effects of sleep quality and family functioning.

**Methods:**

Data were derived from a community-based cross-sectional survey conducted in Gaoping District, Sichuan Province. The Chinese versions of the Neighborhood Environment Walkability Scale-Abbreviated, the 36-Item Short Form Health Survey, the Insomnia Severity Index, and the Family Assessment Device were used to assess the community-built environment, self-rated health, sleep quality, and family functioning. Based on the Structural Equation Model, a structural equation model was constructed, and the weighted least squares mean- and variance-adjusted estimator was used to test the model. After the preliminary confirmatory factor analysis, model fit was evaluated using the comparative fit index, Tucker-Lewis’s index, root mean square error of approximation, and standardized root mean square residual.

**Results:**

This study included 2,705 adults (mean age 51.8 ± 18.3 years; 60% female). After adjusting for socioeconomic characteristics, the community-built environment was not significantly associated with self-rated health (all *p* > 0.05). In contrast, better sleep quality was identified as the strongest predictor of higher self-rated health (*β* = 0.383, *p* < 0.001). The hypothesized serial mediation effect was not statistically significant (*β* = −0.008, *p* = 0.068).

**Conclusion:**

In the mixed urban–rural context of western China, the community-built environment was not significantly associated with the self-rated health among community-dwelling adults. Notably, sleep quality was identified as the predominant factor affecting self-rated health outcomes.

## Graphical abstract

**Figure:**
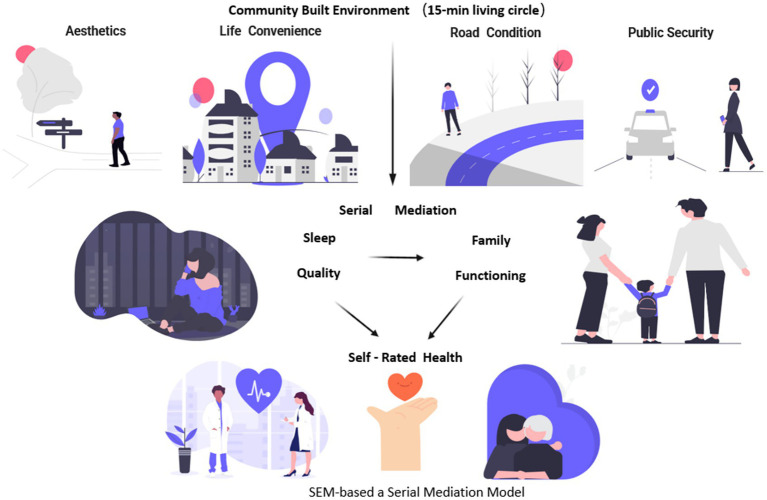

## Highlights

- In the mixed urban–rural context of western China, the impact of the community-built environment on health outcomes was overshadowed by sleep quality and family functioning.
- Methodological rigor was reinforced by using the weighted least squares mean and variance adjusted estimator, ensuring robust structural equation model analysis for ordinal survey data.
- Sleep quality was identified as the strongest independent factor associated with higher self-rated health (*β* = 0.383, *p* < 0.001) among community-dwelling adults.

## 1 Introduction

Self-rated health (SRH) is widely acknowledged as a holistic indicator of an individual’s overall health outcomes, physical, psychological, and social well-being (1, 2). It has been well documented that declining SRH serves as a critical early warning signal for functional impairment and chronic diseases among working-age and older populations (3–5). Despite rising life expectancy in China, a concerning “Health-Survival Paradox” has emerged (6). Recent national surveys indicate that sub-optimal health status (SHS) affects 46% to 52% of urban residents in China, with a higher prevalence observed in inland western regions (7, 8). Therefore, identifying key contextual determinants of SRH to mitigate the disease burden associated with SHS has become an important public health topic in China.

It is well-established that SRH is profoundly influenced by modifiable contextual factors, particularly the community-built environment (9–11). Operationalized in line with China’s national “15-minute living circle” policy, which encompasses aesthetics (12), convenience of living (12), road conditions (13, 14), and public safety (15, 16), supportive physical surroundings are fundamental to residents’ well-being (17). However, despite advancements in urban health research, empirical findings regarding the direct benefits of these environmental features remain highly heterogeneous (10, 18). This inconsistency largely stems from existing literature being predominantly rooted in high-density coastal metropolises in eastern China, while the community-built environments in mid-sized inland cities of western China differ significantly (11, 19–21). Characterized by a unique urban–rural mixture and a high proportion of dispersed, self-built housing in western China, these settings may attenuate the direct effects of the community-built environment on health outcomes.

Furthermore, while the direct associations between community-built environments and health outcomes have been extensively studied (22), the underlying bio-psychosocial mechanisms remain insufficiently understood. It is well-documented that environmental stressors can directly disrupt sleep quality, a core biological recovery process for physical and mental health (11, 16).

Cross-sectional and longitudinal studies among Chinese have further confirmed that sleep disturbance can significantly impair family functioning, the cornerstone of social support in Chinese collectivist culture (18, 23, 24). However, the independent mediating roles of sleep quality and family functioning, as well as their sequential mediating effect on the community-built environment with SRH association, have not been comprehensively verified, especially in the urban–rural mixed context of western China.

To fill this knowledge gap, this study aims to examine the relationships among the community-built environment, sleep quality, family functioning, and SRH among community-dwelling adults in urban-western China. Using Structural Equation Modeling (SEM), we propose two overarching hypotheses:

*Hypothesis 1 (H1)*: A more supportive community-built environment is directly and positively associated with better self-rated health.

*Hypothesis 2 (H2)*: Sleep quality and family functioning sequentially mediate the relationship between the community-built environment and self-rated health.

## 2 Methods

### 2.1 Study design and participants

Between July and August 2023, a cross-sectional study was conducted in urban communities in Gaoping District, Nanchong City, Sichuan Province, China. Ethical approval was obtained from the Institutional Review Boards of the affiliated university (No. 2024019). All participants signed written consent before data collection. Participants were selected using a multistage cluster-randomized design. Five village committees were randomly selected from three townships and two sub-districts. When the number of village committees was insufficient, neighboring committees or villages were merged. 164 households were randomly selected from each village, and a total of 4,100 households were included. One permanent resident aged 18 and above was selected for the nearest birthday in each household. The final valid sample was 2,705. Formula used to compute sample size: $n=\frac{Z_{1-\alpha /2}^{2}\;p\;(1-p)}{d^{2}}$ .

The single proportion formula was used to compute the sample size at a 95% confidence level (Z = 1.96), assuming a prevalence of 10.3% (diabetes in Sichuan Province, 2013). The design effect was set as 2, and the acceptable relative error was 23%. Each stratum should have at least 1,266 individuals. After gender stratification and accounting for 5% non-response, the target sample size was 2,700.

Participants were included if they were community-dwelling adults, had ≥6 months of residency in the community, could complete the structured questionnaire independently or with assistance, and provided informed consent. Exclusion criteria: (1) had a history of psychiatric disorders; (2) acute illness during the survey; or (3) communication difficulties.

### 2.2 Study procedure and data collection

All interviewers received standard training on survey methodology, neutrality, and informed consent. For participants with low literacy, visual impairments, or mobility issues, interviewers read aloud and record verbatim responses during face-to-face interviews. At least 10% of interviews were conducted at participants’ residences when they could not attend the survey. Two researchers independently entered data into Excel software, reconciled them using range and logic checks, then exported them to SPSS 27.0 (IBM Corp., Armonk, NY, USA) and R statistical software version 4.5.1 (R Core Team, 2025) for analysis. Quality control included random back-checks of 5% of households, time-stamp audits, and community code geo-verification.

### 2.3 Variables and measurements

#### 2.3.1 Community-built environment

This study used the validated Chinese version of the Neighbourhood Environment Walkability Scale-Abbreviated (NEWS-A) to assess the community-built environment with a Cronbach’s alpha of 0.907 (25, 26). It contained 17 criteria in four categories: road conditions, aesthetics, traffic, and public safety. From 1 to 5, each item is rated for strong agreement (very good) to strong disagreement (very terrible). Scoring was standardized so that higher scores consistently indicated a more favorable perception of the environment.

#### 2.3.2 Sleep quality

Sleep quality was evaluated using the Insomnia Severity Index (ISI), a widely validated seven-item self-report scale designed to assess sleep difficulties over the past 2 weeks, with a Cronbach’s alpha of 0.957 (1, 27, 28). Each item is rated on a 5-point scale (0–4), with a total score ranging from 0 to 28; higher total score denotes poorer sleep quality. However, for this study and SEM analysis, the total score was reverse-coded, so a higher total score represents better sleep quality.

#### 2.3.3 Family functioning

Family functioning was assessed using the Family Assessment Device (FAD), which evaluates six dimensions of family functioning with a Cronbach’s alpha of 0.827 (29). Each item is rated on a 4-point Likert scale ranging from 1 (strongly agree) to 4 (strongly disagree). Higher scores on the FAD subscales and total score indicate worse family functioning. Data were reverse-coded so higher scores indicate better family functioning.

#### 2.3.4 Self-rated health

The Chinese-validated General Health (GH) subscale of the 36-Item Short Form Health Survey (SF-36) was used to examine self-rated health with a Cronbach’s alpha of 0.813 (30). GH measures an individual’s self-assessment of their health state and trends. A higher score indicates better perceived health. While a lower score indicates worse health. A composite GH score of 1 to 5 is calculated by recording and averaging items, so a higher score consistently indicates better health.

#### 2.3.5 Covariates

Covariates included some sociodemographic variables such as age, gender, marital status, education, residency, and family economic position. The selection of these factors was informed by some previous research and notable correlations identified in initial studies.

### 2.4 Statistical analysis

#### 2.4.1 General statistical procedures

All statistical analyses were performed using R statistical software (Version 4.5.1; R Core Team, 2025). Descriptive statistics were computed for all study variables. Continuous variables were presented as mean ± standard deviation (SD) for normally distributed data, and as median (interquartile range, IQR) for non-normally distributed variables (such as community-built environment scores and sleep quality scores, see Supplementary Table S6, Figure S2). Categorical variables were expressed as frequencies and percentages. Data quality and outlier management were strictly assessed before analysis through a two-step verification process. The Shapiro–Wilk test and Mardia’s test were employed to assess univariate and multivariate normality, respectively.

#### 2.4.2 Correlation and factorability assessment

To explore the interrelationships among exposure and mediating variables, pairwise correlations (Spearman’s rank correlation for ordinal data or Pearson’s for continuous data, as appropriate) were calculated between the community-built environment, family functioning, sleep quality, and self-rated health. The factorability of the community-built environment items was assessed using the Kaiser–Meyer–Olkin (KMO) measure of sampling adequacy and Bartlett’s test of sphericity. A KMO value > 0.80 and a significant Bartlett’s test (*p* < 0.05) were considered indicative of data suitability for factor analysis.

#### 2.4.3 Measurement and SEM

SEM was conducted using the lavaan package to test the hypothesized serial mediation model. The analysis proceeded in two phases:

① Measurement Model: Exploratory Factor Analysis (EFA) was initially conducted to identify the latent structure of the community-built environment scale by using the minimum residual (minres) extraction method with oblimin rotation. Based on the EFA results, latent constructs were defined in the SEM measurement model. ② Structural Model: A serial mediation model was constructed to examine the pathways from community-built environment (Latent Factors) to SRH, with sleep quality and family functioning serving as serial mediators. Covariates included age, gender, marital status, education, family economics, residency, smoking, and drinking frequency.

Given that the community-built environment items were measured on ordinal scales and multivariate normality assumptions were violated (indicated by significant Mardia’s test results), the Weighted Least Squares Mean and Variance adjusted (WLSMV) estimator was utilized, which is robust to non-normality and specifically designed for modeling ordinal data.

Model fit was evaluated using the following indices: the Comparative Fit Index (CFI), the Tucker-Lewis Index (TLI), the Root Mean Square Error of Approximation (RMSEA), and the Standardized Root Mean Square Residual (SRMR). The criteria for acceptable model fit were defined: CFI and TLI ≥ 0.90, RMSEA ≤ 0.08, and SRMR ≤ 0.08. The significance of direct and indirect effects was tested, and 95% confidence intervals (CIs) were reported. A two-tailed *p* < 0.05 was considered statistically significant.

## 3 Results

### 3.1 Description of included participants

Table 1 presents the baseline characteristics of the participants (*N* = 2,705). The mean age was 51.84 ± 18.30 years, with a distribution of 40% male and 60% female. Ethnicity was predominantly Han (99%), with a small proportion (0.6%) identifying as other ethnic groups. Regarding educational attainment, 34% of participants had completed high school or vocational education, while 16% had a bachelor’s degree or higher. In terms of residency, 44% lived in urban areas, 30% in towns, and 27% in rural regions. 67.5% of participants reported an average family economic status. 14% indicated poor family economic status, and 3% indicated very poor family economic status. The average score of activities of daily living (ADL) was 15.08 ± 4.22.

**Table 1:** Baseline characteristics of the study population (*N* = 2,705).

Characteristic	Overall (*N* = 2,705)
Gender (*n*, %)	
Male	1,091 (40%)
Female	1,613 (60%)
Age (years)	51.84 ± 18.30
Ethnicity (*n*, %)	
Han	2,688 (99%)
Others	16 (0.6%)
Marriage (*n*, %)	
Single	345 (13%)
Married	1,994 (74%)
Widows	283 (10%)
Divorced	82 (3.0%)
Education (*n*, %)	
No formal education	409 (15%)
Elementary school or below	575 (21%)
Middle school	358 (13%)
High school/vocational school/technical school	922 (34%)
Bachelor’s degree or above	440 (16%)
No. of family	3.14 ± 1.59
Residency (*n*, %)	
Rural	722 (27%)
Town	804 (30%)
Urban	1,178 (44%)
Family economics (*n*, %)	
Very good	104 (3.8%)
Good	317 (12%)
Average	1,816 (67%)
Poor	385 (14%)
Very poor	82 (3.0%)
Illness	3.34 ± 1.26
Fitness same	2.24 ± 1.11
Worsen	3.10 ± 1.26
Fitness self	2.32 ± 1.11
SF. total	2.99 ± 1.23
Family function	28.89 ± 4.60
Com. enviornment	61.05 ± 12.09
ADL	15.08 ± 4.22
Sleep quality	13.75 ± 6.41

FamFun was family functioning. Com. Env was community-built environment. ADL was activity of daily life. SF. Total means self-rated health total.

Regarding key variables, the average score of SRH was 2.99 ± 1.23 (on a 5-point scale). After reverse-coding, family functioning scored 28.89 ± 4.60, and the mean sleep quality (measured by ISI) score was 13.75 ± 6.41 (out of 28). The community-built environment score was 61.05 ± 12.09.

### 3.2 Histograms of family functioning and sleep quality scores

Supplementary Figure S1 illustrates the distribution of the family functioning scores among the participants. The scores were predominantly clustered around the value of 30, with the highest frequency observed in the 29–31 range. The distribution exhibited a noticeable right skew, with frequencies gradually decreasing as scores deviated from this central tendency towards both lower and higher values. A small number of participants reported scores near 15.

Supplementary Figure S2 represents the distribution of sleep quality (ISI) scores among the participants. The data showed a marked concentration of participants with scores in the range of 5 to 10. The distribution was heavily skewed towards the lower end of the scale, with a gradual decrease in frequency as the scores increased. A minority of participants had scores in the 20–25 range.

### 3.3 Correlations analysis between community-built environment, sleep quality, family functioning, and SRH

Bivariate correlations among the study variables are presented in Table 2. The results showed that the community-built environment was significantly and positively correlated with family functioning (*r* = 0.07, *p* < 0.01) and sleep quality (ISI) (*r* = 0.12, *p* < 0.01).

**Table 2:** Means, standard deviations, and correlations among study variables.

Variables	M	SD	1	2	3	4
Community-built environment	3.61	0.69	—			
Sleep quality (ISI)	1.93	0.89	0.12**	—		
Family functioning	2.42	0.37	0.07**	0.02	—	
Self-rated health (SRH)	2.99	1.23	−0.02	0.15**	0.31**	—

**p < 0.01. M = Mean; SD = Standard Deviation. Means for variables 1–3 represent item averages.

Regarding the outcome variable, self-rated health (SRH) was positively correlated with family functioning (*r* = 0.31, *p* < 0.01) and positively correlated with sleep quality scores (r = 0.15, *p* < 0.01). However, no significant direct correlation was found between the community-built environment and SRH (*r* = −0.02, *p* > 0.05). The relationship between the two mediators (sleep quality and family functioning) was not statistically significant (*r* = 0.02, *p* > 0.05). A complete correlation matrix is provided in Supplementary Tables S2–S5, Figures S3–S6.

### 3.4 SEM modeling

#### 3.4.1 Assessing assumptions and factor structure

The Kaiser–Meyer–Olkin (KMO) measure was 0.920, and Bartlett’s test of sphericity was significant (*x*^2^ = 34933.87, df = 136, *p* < 0.001). Normality tests indicated the assumption of univariate normality (the Shapiro–Wilk test for all items, *p* < 0.05) and multivariate normality (Mardia’s test for skewness, *p* < 0.001, and kurtosis, *p* < 0.001) was violated. Detailed results are provided in Supplementary Table S6.

#### 3.4.2 Correlations between observational variables

Pairwise correlations among the 17 community-built environmental variables (Com. Env) were examined, with coefficients (*r*) ranging from moderate to strong. Specifically, high correlations were observed between items, such as Com. Env16 and Com. Env17 (*r* = 0.894, *p* < 0.001). The complete correlation matrix is presented in the Supplementary material.

#### 3.4.3 Exploratory factor analysis (EFA)

A five-factor solution (MR1 to MR5) was identified for the 17 community-built environment items, explaining 62.0% of the cumulative variance. During the subsequent measurement model validation, one factor (CE2) was excluded from the final model due to suboptimal internal consistency and low factor loadings. The remaining four latent factors (CE1, CE3, CE4, and CE5) were retained for the structural equation modeling. All four latent factors demonstrated acceptable internal consistency. Factor loadings and summary statistics are provided in Supplementary Tables S7, S8.

#### 3.4.4 Structural equation modeling (SEM)

##### 3.4.4.1 Model specification and identification

**Figure 1:**
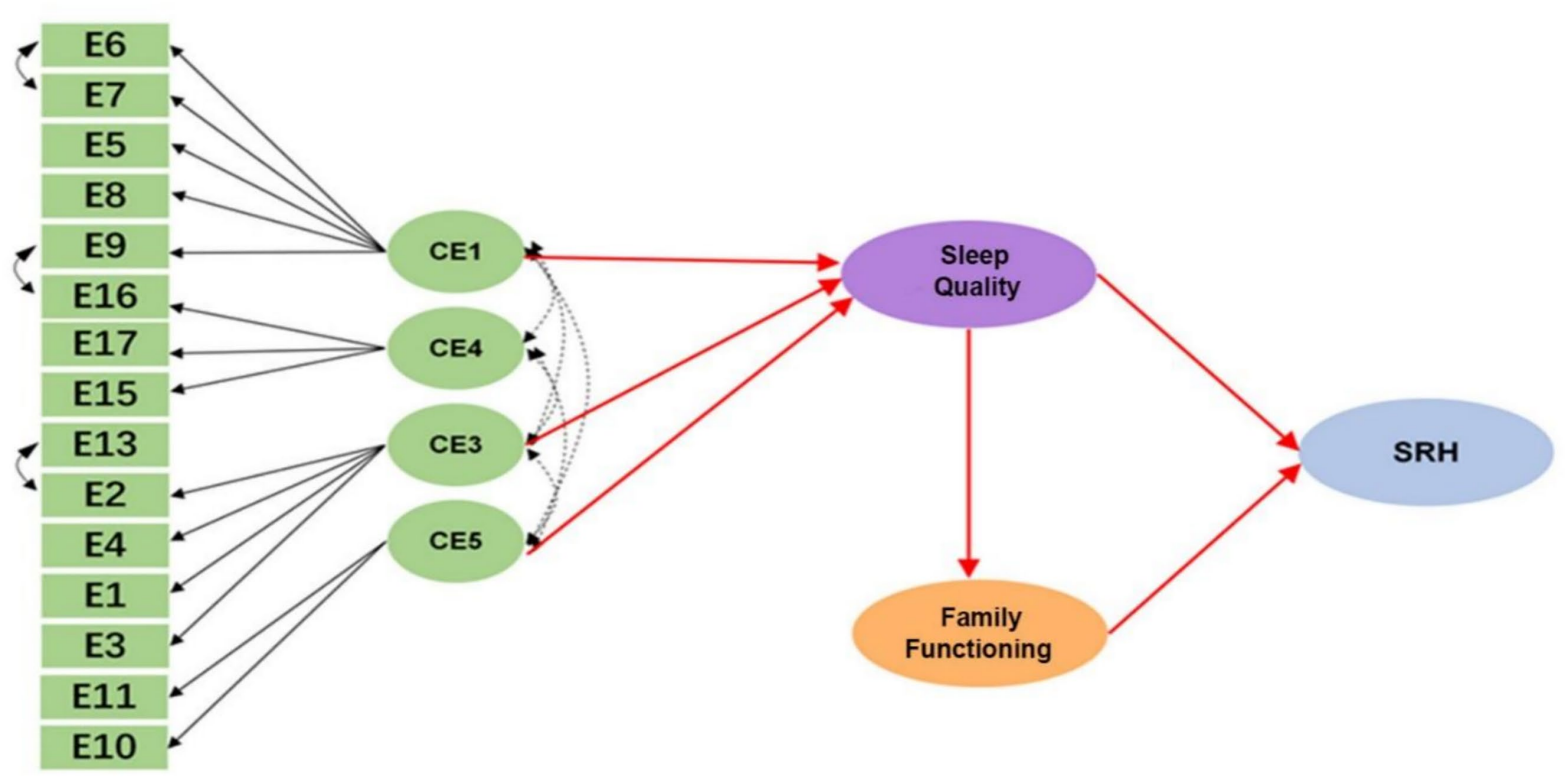
Structural equation model specification.

The final structural model (Figure 1) demonstrated excellent overall fit to the data. Key absolute and incremental fit indices were well within acceptable ranges: CFI = 0.995, TLI = 0.997, RMSEA = 0.080, and RMR = 0.044.

##### 3.4.4.2 Modification

Modification indices suggested minor structural adjustments, as shown in Supplementary Table S9. The final structural model showed excellent overall fit (Table 3), with GFI = 0.994, AGFI = 0.991, CFI = 0.995, and TLI = 0.997. RMSEA was 0.080, and RMR was 0.044.

**Table 3:** Summary of model fit indices and interpretation.

Category	Index	Value
Absolute fit	GFI	0.994
	AGFI	0.991
	RMSEA	0.080
	RMR	0.044
Relative fit	CFI	0.995
	TLI	0.997
	NFI	NA
	IFI	0.995
Parsimony fit	PGFI	0.641
	PNFI	1.704

GFI, Goodness-of-fit index; AGFI, adjusted GFI; RMSEA, root mean square error of approximation; RMR, root mean square residual; SRMR, standardized RMR; CFI, comparative fit index; TLI (NNFI), Tucker–Lewis Index (Non-Normed Fit Index); NFI, normed fit index; IFI, incremental fit index; PGFI, parsimony GFI; PNFI, parsimony NFI.

##### 3.4.4.3 Measurement model

The measurement model defined the relationships between the latent variables and their corresponding observed indicators. Factor loadings for the retained community-built environment factors (CE1, CE3–CE5) were statistically significant (*p* < 0.05), except the relationship between CE5 (community-built environment 5) and the item Com. Env10 (*p* = 0.664). Detailed estimates and statistics are presented in Supplementary Table S10.

##### 3.4.4.4 Structural model

Table 4 presents the path coefficient estimates from the structural model. Sleep quality was positively associated with SRH (est = 0.392, *p* < 0.001). Family functioning showed a significant positive relationship with SRH (est = 0.071, *p* = 0.004). Regarding the mediators, sleep quality was negatively associated with family functioning (est = −0.115, *p* = 0.002). and the family economics was associated with worse sleep quality (est = 0.268, p < 0.001).

**Table 4:** Outcome and predictor statistics.

Outcome	Predictor	B	SE	*Z*	*p*-value	STD	LCI	HCI
SRH	Sleep quality	0.392	0.039	10.025	≤0.001	0.338	0.315	0.468
SRH	Age	0.019	0.003	6.153	≤0.001	0.273	0.013	0.025
SRH	Education	−0.171	0.038	−4.512	≤0.001	−0.181	−0.245	−0.097
SRH	Family economics	0.239	0.043	5.605	≤0.001	0.135	0.156	0.323
SRH	Family functioning	0.071	0.024	2.917	0.004	0.059	0.023	0.118
SRH	Marriage	−0.086	0.064	−1.355	0.175	−0.038	−0.211	0.038
SRH	Drinking freq	0.027	0.02	1.329	0.184	0.036	−0.013	0.066
SRH	Gender	−0.066	0.078	−0.841	0.400	−0.026	−0.219	0.088
SRH	Smoke status	−0.068	0.093	−0.734	0.463	−0.023	−0.251	0.114
SRH	Residency	0.004	0.045	0.099	0.921	0.002	−0.083	0.092
Sleep quality	Family economics	0.268	0.045	5.977	≤0.001	0.176	0.18	0.356
Sleep quality	CE1 (aesthetics)	−0.108	0.178	−0.61	0.542	−0.104	−0.457	0.24
Sleep quality	Residency	0.168	0.050	3.385	0.001	0.102	0.071	0.265
Sleep quality	CE5 (security)	−0.098	0.199	−0.491	0.624	−0.093	−0.487	0.292
Sleep quality	Drinking freq	−0.024	0.021	−1.177	0.239	−0.038	−0.064	0.016
Sleep quality	Gender	0.08	0.074	1.08	0.28	0.037	−0.065	0.226
Sleep quality	Age	−0.002	0.003	−0.678	0.498	−0.032	−0.007	0.004
Sleep quality	Education	0.022	0.037	0.601	0.548	0.027	−0.05	0.095
Sleep quality	Marriage	0.037	0.055	0.677	0.499	0.019	−0.071	0.145
Sleep quality	CE3 (traffic)	−0.007	0.048	−0.144	0.885	−0.007	−0.101	0.087
Sleep quality	Smoke status	−0.004	0.092	−0.047	0.962	−0.002	−0.184	0.175
Family functioning	Sleep quality	−0.115	0.037	−3.124	0.002	−0.119	−0.188	−0.043
Family functioning	Age	−0.005	0.003	−1.656	0.098	−0.087	−0.011	0.001
Family functioning	Gender	0.156	0.073	2.138	0.033	0.074	0.013	0.300
Family functioning	Residency	0.09	0.038	2.364	0.018	0.056	0.015	0.165
Family functioning	Family economics	0.074	0.034	2.18	0.029	0.05	0.008	0.141
Family functioning	Marriage	0.063	0.082	0.767	0.443	0.033	−0.098	0.224
Family functioning	Drinking freq	0.013	0.019	0.686	0.493	0.021	−0.024	0.05
Family functioning	Smoke status	−0.034	0.087	−0.389	0.697	−0.013	−0.205	0.137
Family functioning	Education	0.002	0.044	0.052	0.959	0.003	−0.083	0.088

B, unstandardized regression coefficient; SE, standard error; Z, Wald z-statistic; STD, standardized regression coefficient; LCI, lower limit of 95% confidence interval; HCI, higher limit of 95% confidence interval.

The path coefficients from community-built environment factors (CE1, CE3, CE5) to sleep quality were not statistically significant (all *p* > 0.05). Hypothesis testing results were summarized as follows, Hypothesis 1 and Hypothesis 2 were not supported.

##### 3.4.4.5 Direct and indirect effect estimates

Table 5 presents the estimates for the direct, indirect, and total effects within the structural model, reflecting the relationships among community-built environment factors (CE1, CE3, CE5), sleep quality, family functioning, and SRH.

**Table 5:** Effect estimates and statistics.

Effect	B	SE	Z	*p*-value	LCI	HCI
Direct effects						
Dir_CE1_SRH	<0.001	<0.001	NA	NA	<0.001	<0.001
Dir_CE3_SRH	<0.001	<0.001	NA	NA	<0.001	<0.001
Dir_CE5_SRH	<0.001	<0.001	NA	NA	<0.001	<0.001
Indirect effects (Chain)						
ind2_CE1_Chain_SRH	0.001	0.002	0.572	0.567	−0.002	0.004
ind2_CE3_Chain_SRH	<0.001	<0.001	0.143	0.886	−0.001	0.001
ind2_CE5_Chain_SRH	0.001	0.002	0.48	0.631	−0.002	0.004
Indirect effects (simple)						
ind_CE1_Sleep_SRH	−0.042	0.07	−0.606	0.545	−0.180	0.095
ind_CE3_Sleep_SRH	−0.003	0.019	−0.144	0.886	−0.040	0.034
ind_CE5_Sleep_SRH	−0.038	0.078	−0.492	0.623	−0.190	0.114
ind_Sleep_Fam_SRH	−0.008	0.004	−1.827	0.068	−0.017	0.001
Total indirect effects						
ind_total_CE1_SRH	−0.042	0.069	−0.606	0.545	−0.176	0.093
ind_total_CE3_SRH	−0.003	0.018	−0.144	0.886	−0.039	0.034
ind_total_CE5_SRH	−0.037	0.076	−0.492	0.623	−0.186	0.112
Effects on mediators						
te_CE1_on_FamFun	0.013	0.021	0.595	0.552	−0.029	0.054
te_CE3_on_FamFun	0.001	0.006	0.144	0.886	−0.01	0.012
te_CE5_on_FamFun	0.011	0.023	0.488	0.625	−0.034	0.056
Total effects						
tot_CE1_on_SRH	−0.042	0.069	−0.606	0.545	−0.176	0.093
tot_CE3_on_SRH	−0.003	0.018	−0.144	0.886	−0.039	0.034
tot_CE5_on_SRH	−0.037	0.076	−0.492	0.623	−0.186	0.112
tot_Sleep_on_SRH	0.383	0.038	9.971	<0.001	0.308	0.459

B, unstandardized regression coefficient; SE, standard error; Z, Wald z-statistic; STD, standardized regression coefficient; LCI, lower limit of 95% confidence interval; HCI, higher limit of 95% confidence interval; Dir: direct effects; ind: indirect effects; tot: total effects.

Community-built environment factors (CE1, CE3, CE5) demonstrated no significant direct or total effect on SRH (*p* > 0.05). The analysis indicated a marginally significant indirect path from sleep quality to SRH via family functioning (est ≈ −0.008, *p* = 0.068). Crucially, Sleep Quality exhibited a strong and highly significant total effect on SRH (est = 0.383, *p* < 0.001).

## 4 Discussion

With a specific focus on exploring the community-built environment may influence SRH among community-dwelling adults, and by incorporating key psychosocial factors, this study proposed a latent serial mediation with an SEM model to link environmental perceptions, sleep quality, family functioning, and SRH, based on a representative sample of 2,705 adults. In the urban–rural mixed context of western China, no significant direct or indirect association between community-built environment and SRH was observed. In contrast, sleep quality and family functioning were identified as the dominant predictors of SRH, and the hypothesized serial mediation pathway from community-built environment to SRH via sleep quality and family functioning was not statistically supported.

### 4.1 Association between community-built environment and SRH

It has been well-documented that self-rated health (SRH) can be significantly promoted by supportive community-built environments globally (31, 32), with established pathways including reduced environmental stressors, improved sleep quality, and enhanced community social support (33). However, profound geographical and cultural heterogeneity has been identified in the health effects of the community-built environment; well-verified pathways in developed regions and high-density metropolises in eastern China have been found to be significantly attenuated in mid-sized cities of western China characterized by urban–rural mixed contexts (22, 34, 35).

In the present study, no significant direct association between the perceived community-built environment and SRH was observed, nor was a significant link with sleep quality identified (all *p* > 0.05), and thus Hypothesis 1 and Hypothesis 2 were not supported. This null finding does not contradict established environmental health theories but is driven by the unique residential structure of the study cohort, which was composed of 44% urban, 30% town, and 27% rural residents. High-density urban communities in this region are characterized by a lack of sleep-friendly public facilities, while dispersed self-built housing in rural and town areas is associated with insufficient standardized community services (36–38). Residents’ evaluation of the community-built environment has been found to be primarily focused on daily life convenience (12), with extremely low dependence on community-level built environment features, which fundamentally weaken its health effects.

### 4.2 Sleep quality as the strongest predictor of SRH

It has been well-documented that sleep quality is a strong global predictor of self-rated health (SRH), with sleep disturbance identified as an independent risk factor for poor subjective health, emotional distress, functional impairment, and chronic disease onset (1, 6). A recent meta-analysis has revealed that individuals with inadequate sleep have a 2-fold higher risk of poor SRH (OR ≈ 2.10), with population-based studies in China further verifying this association in inland community-dwelling populations (39–41). The health-protective effects of high-quality sleep are well-established across diverse populations, with core pathways including improved emotional regulation, buffered chronic stress, and preserved physical and social functioning (42–44).

In the present study, a significant positive association between better sleep quality and higher SRH was observed (*β* = 0.383, *p* < 0.001). Sleep quality was identified as the strongest independent predictor of SRH in this cohort, with a substantially larger effect size than all other individual and contextual factors included in the model. This finding aligns with extensive prior epidemiological evidence and extends the established sleep-SRH association to the understudied urban–rural mixed population in western China (34, 45).

The dominant effect of sleep quality in this cohort is linked to the unique context of western inland China, where the health impact of the community-built environment is significantly attenuated and formal community support is insufficient. Sleep health, as a core modifiable biological resource, plays a more critical role in residents’ well-being here than in eastern high-density metropolises, with a marginally significant indirect pathway to SRH via family functioning also observed (*β* ≈ 0.008, *p* = 0.068).

### 4.3 Family functioning, sleep-family association, and serial mediation effect

In this study, family functioning was specified as the second mediator linking community-built environment to SRH. A significant positive association between better family functioning and higher SRH was observed in the final adjusted model (*β* = 0.071, *p* = 0.004), consistent with our pre-specified hypothesis, and the effect remained stable after covariate adjustment, confirming its independent protective role on subjective health. In Chinese collectivist culture, especially in inland regions with limited formal community social services, family is the primary source of emotional and practical support for community-dwelling adults (18, 23, 46, 47). National survey data have consistently shown that stable family ties buffer stress-related health damage and enhance psychological security (48, 49, 38), and the high family cohesion observed in this cohort has been suggested to improve SRH by fostering consistent healthy routines and alleviating chronic stress (39, 40).

Our results revealed a significant negative association between sleep quality and family functioning (*β* = −0.115, *p* = 0.002). This inverse relationship may be attributed to unmeasured confounding covariates commonly observed in epidemiological studies (50, 51). The unadjusted bivariate correlation between sleep quality and family functioning was non-significant (*r* = 0.02, *p* > 0.05). A significant negative trend was observed after adjusting for age, family economic status, and other covariates. Additionally, the other association might be partially influenced by potential confounding effects.

Furthermore, within the surveyed population, lower family functioning may reduce participation in family activities, thus leaving more time for rest and thereby improving sleep quality. As a result, sleep quality is enhanced. The core finding is further supported by this behavioral mechanism. It reinforces that self-rated health is predominantly determined by sleep quality.

The hypothesized serial mediation pathway from community-built environment to SRH via sleep quality and family functioning was not statistically supported (*β* = −0.008, *p* = 0.068), with a marginally significant indirect trend from sleep quality to SRH via family functioning observed, which did not meet the *p* < 0.05 significance threshold. The failure of this hypothesized pathway may be driven by the non-significant association between community-built environment and the first mediator, sleep quality, which broke the sequential chain from environmental exposure to health outcomes, aligning with the finding that the community-built environment’s health effect is fundamentally attenuated in this urban–rural mixed context.

### 4.4 Strengths and limitations

While our study provides valuable insights, several limitations should be acknowledged. First, a cross-sectional design limits the ability to establish causal relationships, and longitudinal studies are needed to confirm these causal links. Apart from the community-built environment, self-rated health outcomes may also be influenced by other factors, such as clinical treatment modalities.

Despite these limitations, our study has notable strengths. It is based on a large community-dwelling sample in an understudied western Chinese city and uses structural equation modelling to examine direct and indirect pathways simultaneously, while adjusting for socioeconomic and demographic covariates. This study also provides new localized evidence on the community health mechanisms of self-rated health in urban–rural mixed contexts.

## 5 Conclusion

In summary, our findings reveal that the community-built environment was not significantly associated with the self-rated health (SRH) of community-dwelling adults in western China. Based on a representative sample of 2,705 individuals, we identified sleep quality as the predominant factor driving SRH, with family functioning also playing a supportive role. These findings suggest that targeted interventions aimed at improving sleep quality and strengthening family-based support systems may effectively improve residents’ SRH in this specific context.

## Data Availability

The raw data supporting the conclusions of this article will be made available by the authors, without undue reservation.
