# Supplementary material for: Community-built environment and self-rated health in Western China: a latent serial mediation within SEM of sleep quality and family functioning
Source: Front Public Health. 2026 Apr 24;14:1787068. doi: 10.3389/fpubh.2026.1787068 (PMC13152844; doi:10.3389/fpubh.2026.1787068)
Supplement: Supplementary file 1 [file Table_1.docx]

**Supplementary**

**Supplementary Table S1:** **Baseline characteristics of the study population**3.1 Description of included participants

Table S1 presents the baseline characteristics of the study participants (N = 2,705).

**Table S1. Baseline characteristics of the study population (N = 2,705)**

| Characteristic | Overall (N = 2,705) |
| --- | --- |
| Gender (n, %) |  |
| Male | 1,091 (40%) |
| Female | 1,613 (60%) |
| Age (years) | 51.84 ± 18.30 |
| Ethnicity (n, %) |  |
| Han | 2,688 (99%) |
| Others | 16 (0.6%) |
| Marriage (n, %) |  |
| Single | 345 (13%) |
| Married | 1,994 (74%) |
| Widows | 283 (10%) |
| Divorced | 82 (3.0%) |
| Education (n, %) |  |
| No Formal Education | 409 (15%) |
| Elementary School or Below | 575 (21%) |
| Middle School | 358 (13%) |
| High School/Vocational School/Technical School | 922 (34%) |
| Bachelor’s Degree or Above | 440 (16%) |
| Height (cm) | 159.09 ± 8.67 |
| Weight (kg) | 63.96 ± 19.13 |
| Waist (cm) | 78.62 ± 18.01 |
| SBP (mmHg) | 121.82 ± 24.49 |
| DBP (mmHg) | 82.75 ± 17.52 |
| Illness | 3.34 ± 1.26 |
| Fitness.same | 2.24 ± 1.11 |
| Worsen | 3.10 ± 1.26 |
| Fitness.self | 2.32 ± 1.11 |
| SF.Total | 2.99 ± 1.23 |
| No.of.family | 3.14 ± 1.59 |
| Residency (n, %) |  |
| Rural | 722 (27%) |
| Twon | 804 (30%) |
| Urban | 1,178 (44%) |
| Family.economics (n, %) |  |
| Very Good | 104 (3.8%) |
| Good | 317 (12%) |
| Average | 1,816 (67%) |
| Poor | 385 (14%) |
| Very Poor | 82 (3.0%) |
| FamFun1 | 2.92 ± 0.89 |
| FamFun2 | 1.76 ± 0.68 |
| FamFun3 | 2.78 ± 0.84 |
| FamFun4 | 2.00 ± 0.73 |
| FamFun5 | 2.73 ± 0.82 |
| FamFun6 | 1.92 ± 0.72 |
| FamFun7 | 3.02 ± 0.82 |
| FamFun8 | 2.04 ± 0.74 |
| FamFun9 | 2.93 ± 0.77 |
| FamFun10 | 1.90 ± 0.68 |
| FamFun11 | 3.10 ± 0.80 |
| FamFun12 | 1.79 ± 0.67 |
| Com.Env1 | 4.07 ± 1.05 |
| Com.Env2 | 3.87 ± 1.18 |
| Com.Env3 | 3.36 ± 1.38 |
| Com.Env4 | 3.76 ± 1.12 |
| Com.Env5 | 3.70 ± 1.02 |
| Com.Env6 | 3.72 ± 1.00 |
| Com.Env7 | 3.75 ± 1.05 |
| Com.Env8 | 3.88 ± 0.98 |
| Com.Env9 | 3.53 ± 1.11 |
| Com.Env10 | 3.63 ± 1.08 |
| Com.Env11 | 3.50 ± 1.06 |
| Com.Env12 | 2.50 ± 1.15 |
| Com.Env13 | 3.32 ± 1.04 |
| Com.Env14 | 2.73 ± 1.16 |
| Com.Env15 | 3.87 ± 0.98 |
| Com.Env16 | 3.97 ± 0.97 |
| Com.Env17 | 3.89 ± 0.99 |
| Smoke.bin (n, %) | 602 (22%) |
| Drinking.Freq (n, %) |  |
| 1 | 124 (4.6%) |
| 2 | 104 (3.8%) |
| 3 | 102 (3.8%) |
| 4 | 161 (6.0%) |
| 5 | 226 (8.4%) |
| 6 | 266 (9.8%) |
| 7 | 1,721 (64%) |
| Heav.Drin.Freq | 2.46 ± 4.98 |
| PhyAct.High.Bin (n, %) |  |
| 1 | 226 (13%) |
| 2 | 1,518 (87%) |
| PhyAct.High.Freq | 2.12 ± 1.24 |
| PhyAct.High.Hour |  |
| 0 | 104 (46) |
| 1 | 110 (49) |
| 2 | 10 (4.4) |
| 3 | 2 (0.9) |
| PhyAct.High.Min | 16.19 ± 13.77 |
| PhyAct.Mediun.Bin (n, %) |  |
| 1 | 637 (37%) |
| 2 | 1,107 (63%) |
| PhyAct.Mediun.Freq | 2.92 ± 1.86 |
| PhyAct.Mediun.Hour | 0.63 ± 0.67 |
| PhyAct.Mediun.Min | 17.30 ± 13.78 |
| PhyAct.Walk.Bin (n, %) |  |
| 1 | 1,417 (81%) |
| 2 | 327 (19%) |
| PhyAct.Walk.Freq | 4.78 ± 2.11 |
| PhyAct.Walk.Hour | 0.70 ± 0.80 |
| PhyAct.Walk.Min | 19.40 ± 14.24 |
| PhyAct.Seat.Hour | 4.47 ± 3.56 |
| PhyAct.Seat.Min | 13.49 ± 15.10 |
| ADL1 | 1.04 ± 0.27 |
| ADL2 | 1.03 ± 0.23 |
| ADL3 | 1.06 ± 0.36 |
| ADL4 | 1.06 ± 0.34 |
| ADL5 | 1.06 ± 0.33 |
| ADL6 | 1.04 ± 0.30 |
| ADL7 | 1.18 ± 0.62 |
| ADL8 | 1.07 ± 0.39 |
| ADL9 | 1.09 ± 0.42 |
| ADL10 | 1.10 ± 0.45 |
| ADL11 | 1.08 ± 0.42 |
| ADL12 | 1.14 ± 0.54 |
| ADL13 | 1.04 ± 0.30 |
| ADL14 | 1.09 ± 0.39 |
| Sleep. quality1 | 1.91 ± 1.04 |
| Sleep. quality 2 | 1.90 ± 1.04 |
| Sleep. quality 3 | 1.86 ± 1.00 |
| Sleep. quality 4 | 2.38 ± 1.13 |
| Sleep. quality 5 | 1.90 ± 0.98 |
| Sleep. quality 6 | 1.88 ± 1.00 |
| Sleep. quality 7 | 1.93 ± 1.04 |
| Family functioning | 28.89 ± 4.60 |
| Com.Env | 61.05 ± 12.09 |
| ADL | 15.08 ± 4.22 |
| Sleep Quality | 13.75 ± 6.41 |

FamFun was family functioning.Com.Env was community-built environment; ADL was activity daily life.

3.2 Histograms of Family Functioning and Sleep Quality Scores

Figure S1 illustrates the distribution of the family functioning scores among the participants.


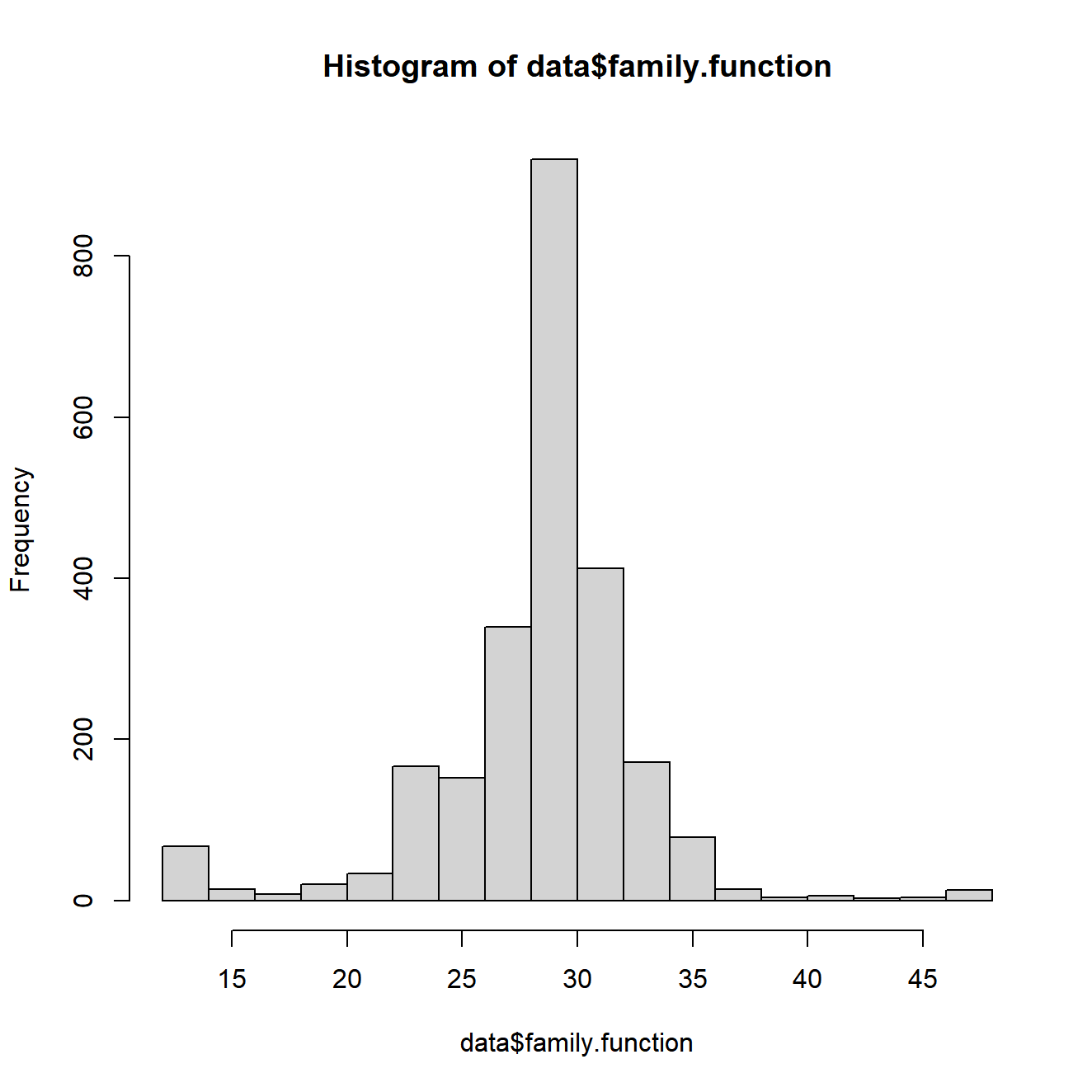


**Figure S1. Histogram of Family Functioning Scores**

Figure S2 represents the distribution of sleep quality scores among the participants.


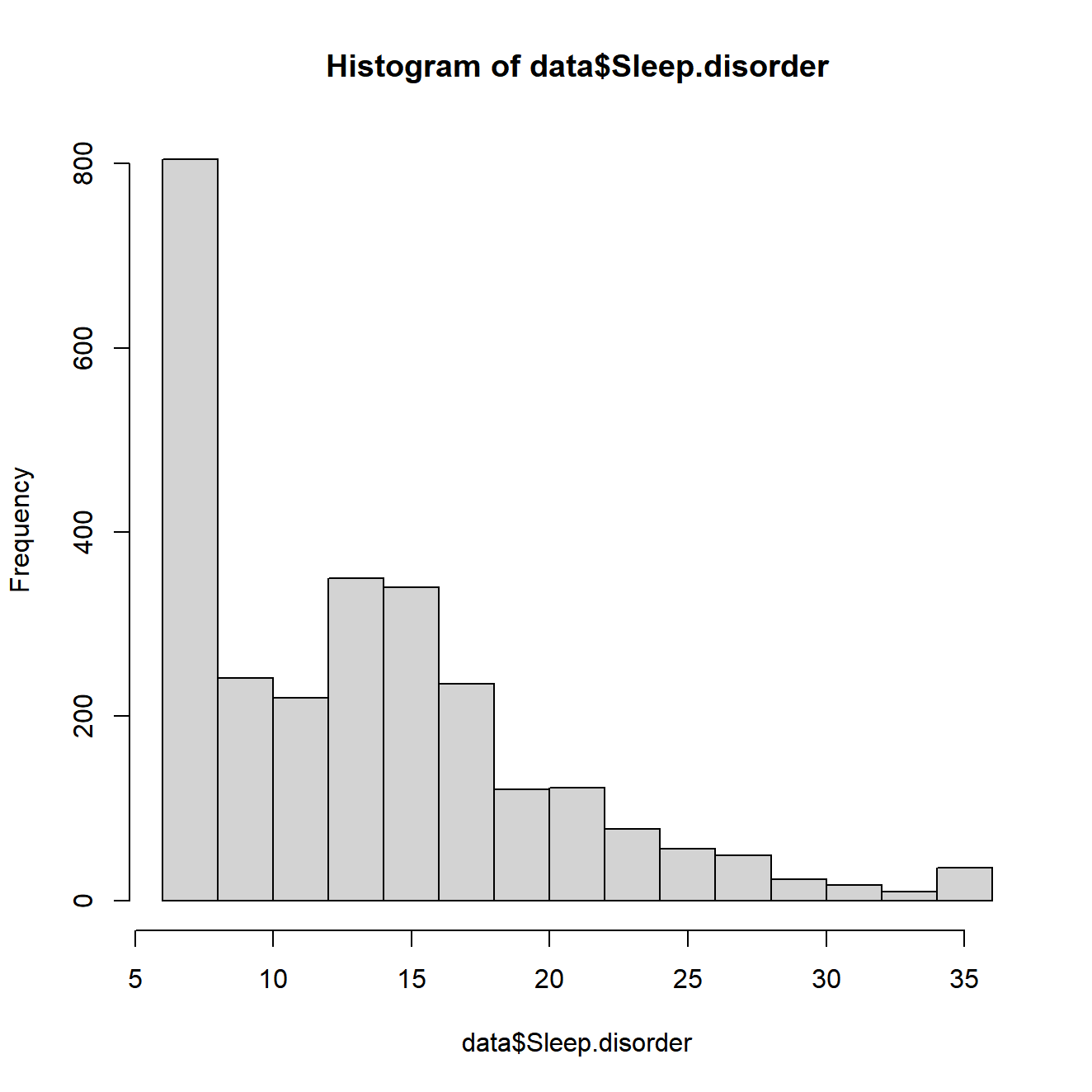


**Figure S2: Histogram of Sleep Quality Scores**

3.3 Correlation analysis between exposures, mediators, and outcomes

3.3.1 Exposure – M1

Table S2 and Figure S3 present the pairwise correlations between family functioning and the 17 community-built environment variables.

**Table S2. Pairwise correlations between community-built environmental and family functioning**

| **Variable 1** | **Variable 2** | ***r*** | ***P* value** |
| --- | --- | --- | --- |
| Enviroment.total.score | family.functioning | 0.064 | 0.023 |
| Com.Env1 | family.functioning | 0.111 | 0.000 |
| Com.Env2 | family.functioning | 0.111 | 0.000 |
| Com.Env3 | family.functioning | 0.049 | 0.085 |
| Com.Env4 | family.functioning | 0.074 | 0.009 |
| Com.Env5 | family.functioning | 0.050 | 0.078 |
| Com.Env6 | family.functioning | 0.061 | 0.032 |
| Com.Env7 | family.functioning | 0.052 | 0.064 |
| Com.Env8 | family.functioning | 0.074 | 0.009 |
| Com.Env9 | family.functioning | 0.038 | 0.178 |
| Com.Env10 | family.functioning | 0.055 | 0.051 |
| Com.Env11 | family.functioning | 0.001 | 0.984 |
| Com.Env12 | family.functioning | -0.061 | 0.030 |
| Com.Env13 | family.functioning | 0.068 | 0.017 |
| Com.Env14 | family.functioning | -0.066 | 0.020 |
| Com.Env15 | family.functioning | 0.042 | 0.139 |
| Com.Env16 | family.functioning | 0.053 | 0.061 |
| Com.Env17 | family.functioning | 0.044 | 0.124 |


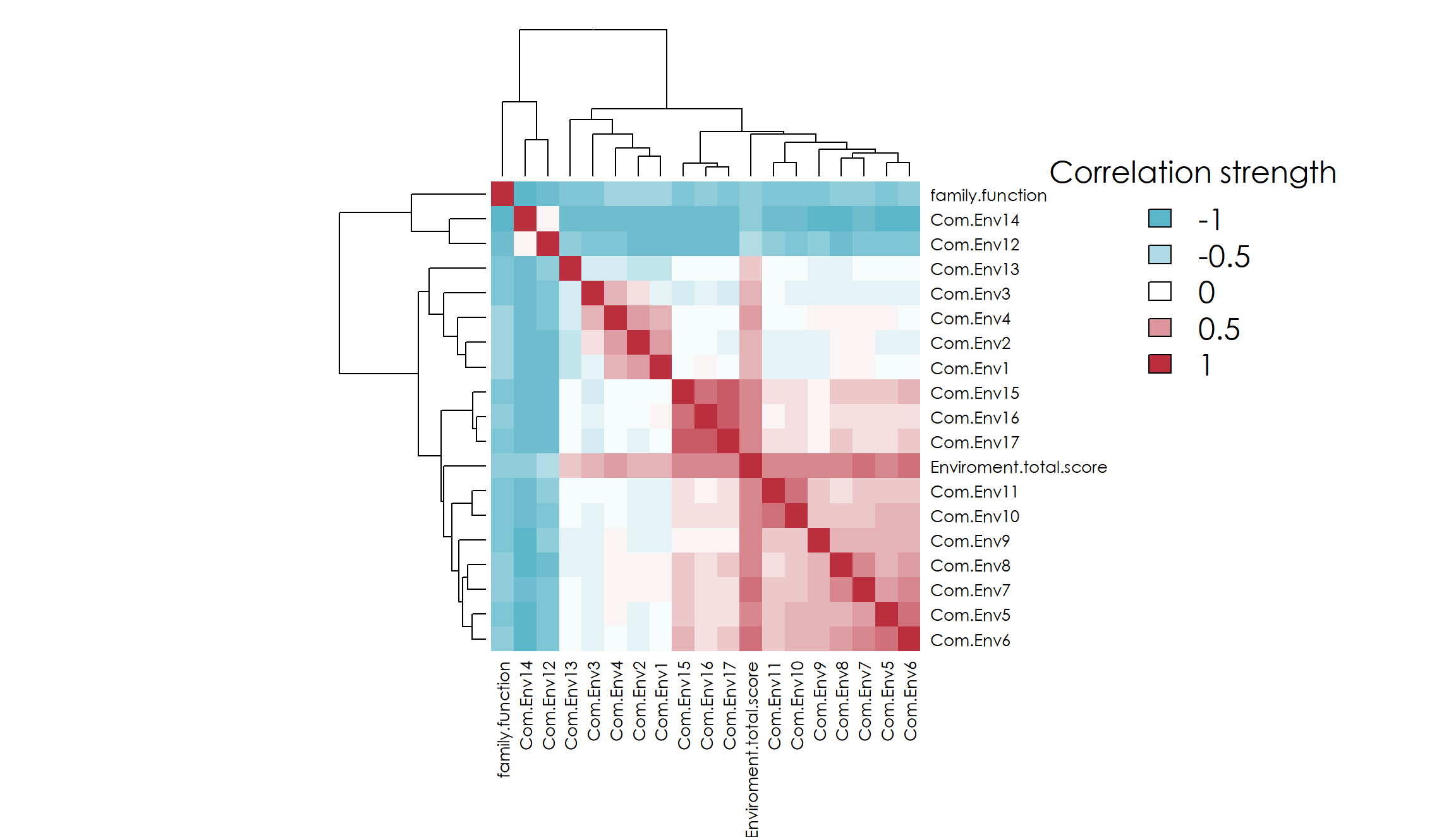


Figure S3. Correlation Heatmap of Family Functioning and Community-built Environment

3.3.2 Exposure - M2

Table S3 and Figure S4 display the pairwise correlations between family functioning and the 17 community-built environment variables.

**Table S3. Pairwise correlations between community-built environment and sleep quality**

| **Variable 1** | **Variable 2** | ***r*** | ***P* value** |
| --- | --- | --- | --- |
| Enviroment.total.score | Sleep.quality | -0.153 | 0.000 |
| Com.Env1 | Sleep.quality | -0.115 | 0.000 |
| Com.Env2 | Sleep.quality | -0.107 | 0.000 |
| Com.Env3 | Sleep.quality | -0.029 | 0.277 |
| Com.Env4 | Sleep.quality | -0.078 | 0.004 |
| Com.Env5 | Sleep.quality | -0.153 | 0.000 |
| Com.Env6 | Sleep.quality | -0.161 | 0.000 |
| Com.Env7 | Sleep.quality | -0.134 | 0.000 |
| Com.Env8 | Sleep.quality | -0.175 | 0.000 |
| Com.Env9 | Sleep.quality | -0.107 | 0.000 |
| Com.Env10 | Sleep.quality | -0.148 | 0.000 |
| Com.Env11 | Sleep.quality | -0.144 | 0.000 |
| Com.Env12 | Sleep.quality | 0.128 | 0.000 |
| Com.Env13 | Sleep.quality | -0.106 | 0.000 |
| Com.Env14 | Sleep.quality | 0.175 | 0.000 |
| Com.Env15 | Sleep.quality | -0.162 | 0.000 |
| Com.Env16 | Sleep.quality | -0.140 | 0.000 |
| Com.Env17 | Sleep.quality | -0.163 | 0.000 |


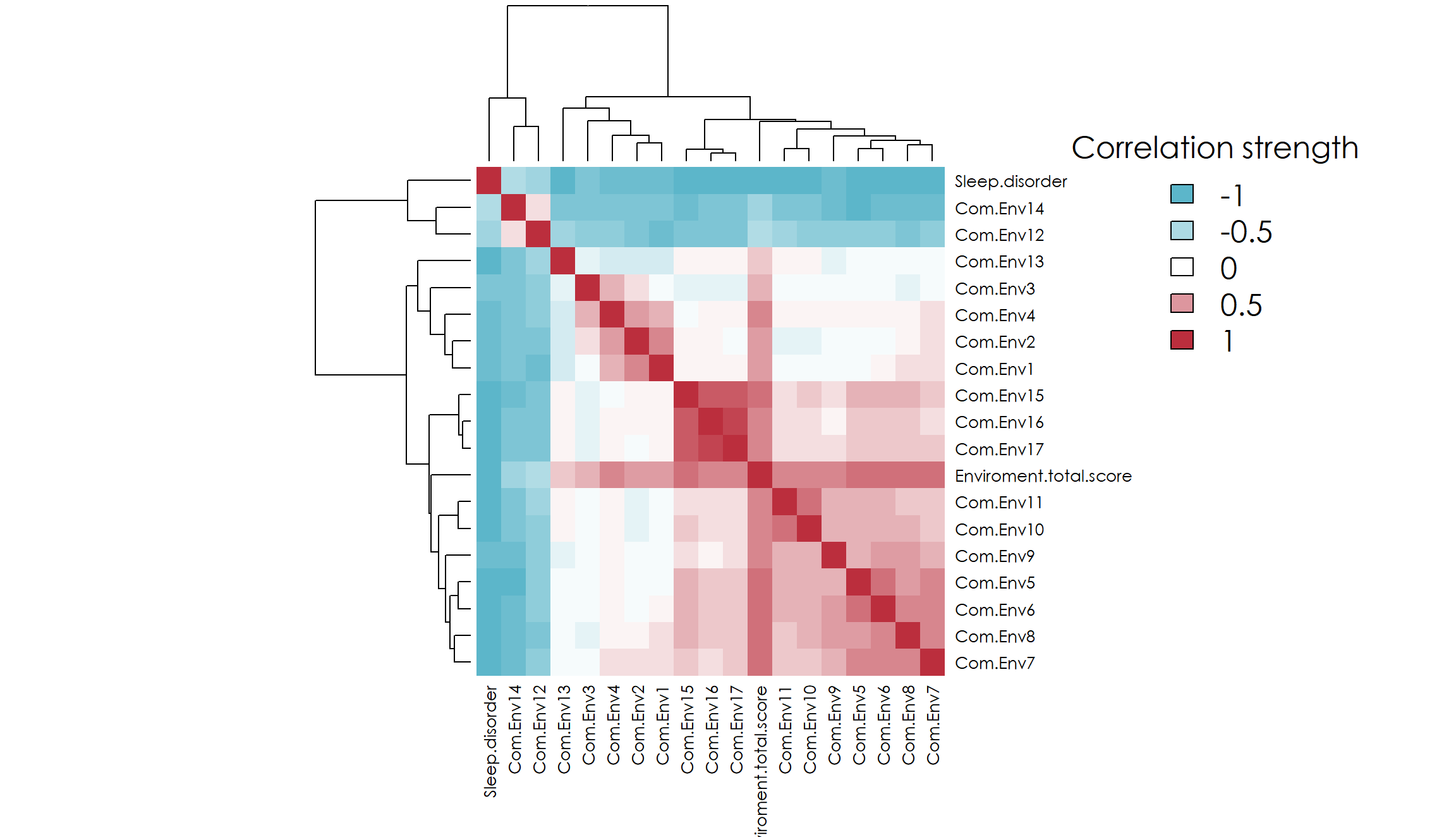


**Figure S4. Correlation Heatmap of Sleep Quality and Community-built Environment.**

3.3.3 Correlation between M1 and M2

Table S4 and Figure S5 show the pairwise correlation between Sleep quality and Family Functioning.

**Table S4. Pairwise Correlation Between Sleep Quality and Family Functioning**

| **Variable 1** | **Variable 2** | ***r*** | ***P* value** |
| --- | --- | --- | --- |
| Sleep.quality | family.functioning | 0.019 | 0.343 |


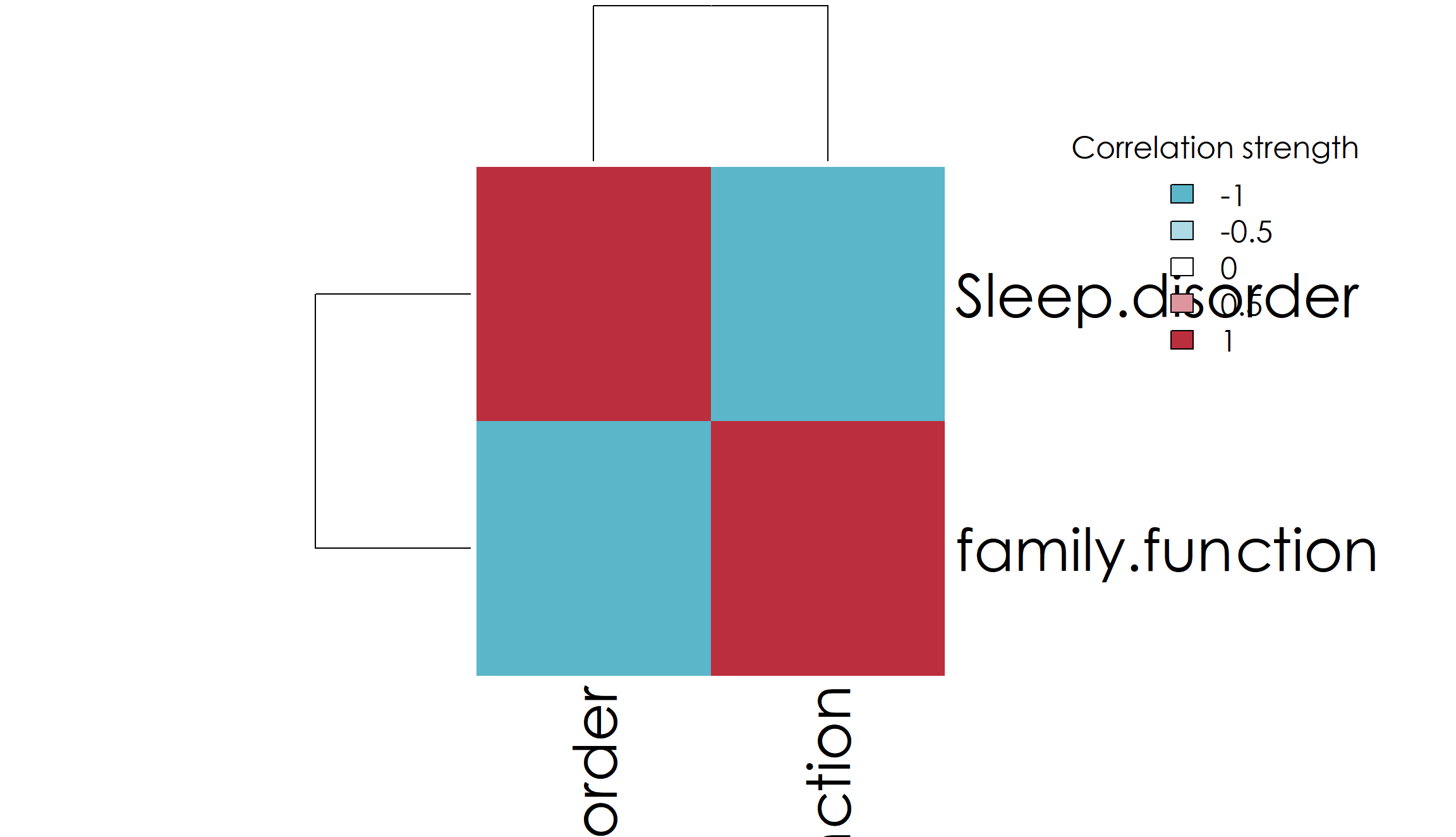


**Figure S5. Correlation Heatmap of Sleep Quality and Family Functioning**

3.3.4 Exposure – Y – Self-rated Health

Table S5 and Figure S6 illustrate the pairwise correlations between SRH (SF.Total) and the 17 community-built environment variables

**Table S5. Pairwise correlations between SF.Total and environmental variables**

| **Variable 1** | **Variable 2** | ***r*** | ***P* value** |
| --- | --- | --- | --- |
| SF.Total | Com.Env1 | -0.020 | 0.463 |
| SF.Total | Com.Env2 | -0.043 | 0.108 |
| SF.Total | Com.Env3 | -0.086 | 0.001 |
| SF.Total | Com.Env4 | -0.026 | 0.343 |
| SF.Total | Com.Env5 | -0.133 | 0.000 |
| SF.Total | Com.Env6 | -0.072 | 0.008 |
| SF.Total | Com.Env7 | -0.064 | 0.018 |
| SF.Total | Com.Env8 | -0.086 | 0.001 |
| SF.Total | Com.Env9 | -0.031 | 0.249 |
| SF.Total | Com.Env10 | -0.107 | 0.000 |
| SF.Total | Com.Env11 | -0.082 | 0.002 |
| SF.Total | Com.Env12 | 0.115 | 0.000 |
| SF.Total | Com.Env13 | -0.167 | 0.000 |
| SF.Total | Com.Env14 | 0.122 | 0.000 |
| SF.Total | Com.Env15 | -0.104 | 0.000 |
| SF.Total | Com.Env16 | -0.095 | 0.000 |
| SF.Total | Com.Env17 | -0.077 | 0.004 |


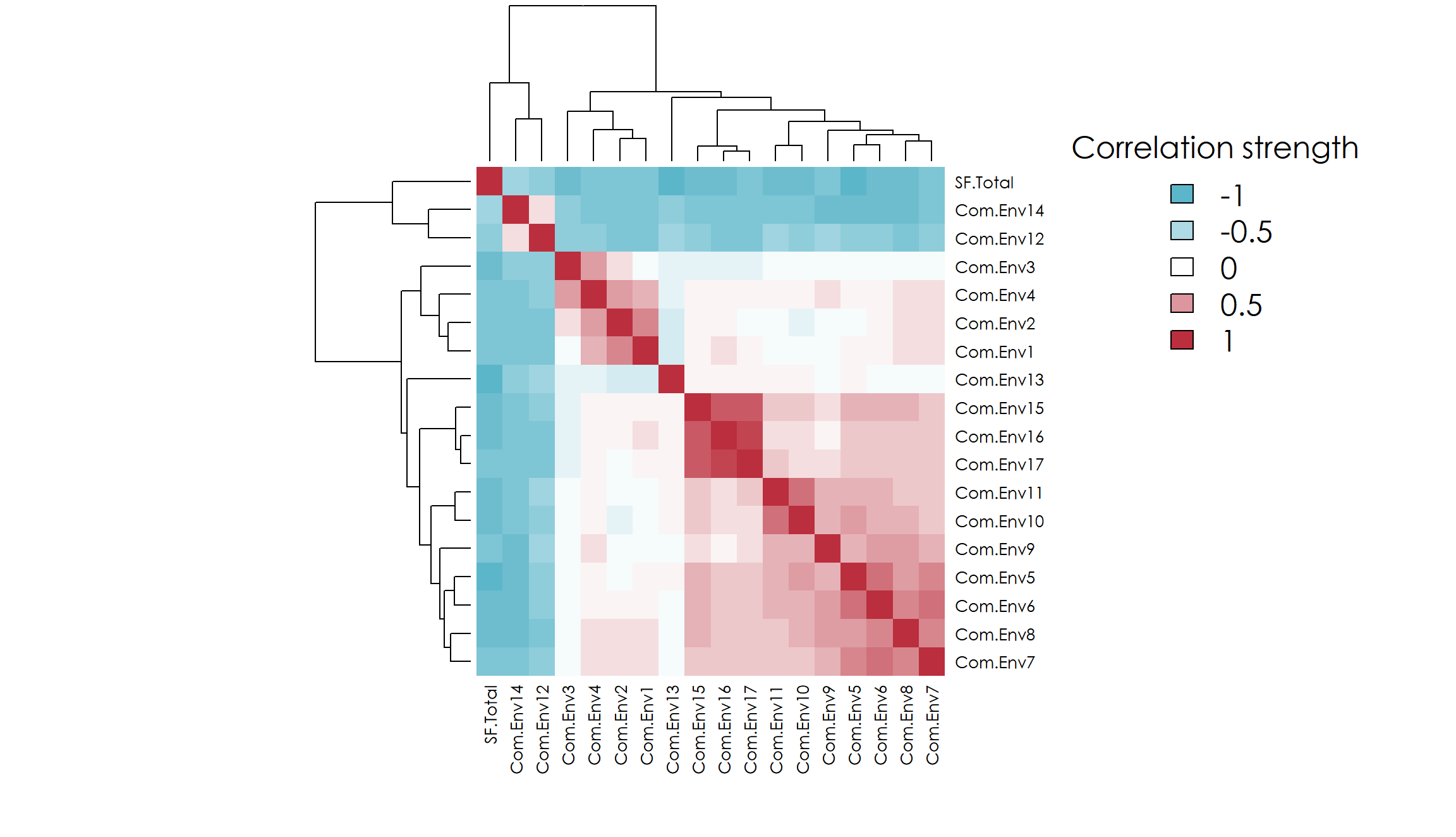


**Figure S6. Correlation Heatmap of SF.Total and Environmental Factors (Com.Env)**

3.4 SEM modeling

3.4.1 Assessing assumptions

Table S6 presents the results of the Shapiro–Wilk test for univariate normality. As indicated in the table, all 17 environmental variables significantly deviated from a normal distribution (P < 0.001).

**Table S6. Shapiro–Wilk Test for Univariate Normality of Environmental Variables**

| **Variable** | **W** | ***P*-value** | **Normality** |
| --- | --- | --- | --- |
| Com.Env1 | 0.801 | <0.001 | No |
| Com.Env2 | 0.830 | <0.001 | No |
| Com.Env3 | 0.875 | <0.001 | No |
| Com.Env4 | 0.864 | <0.001 | No |
| Com.Env5 | 0.877 | <0.001 | No |
| Com.Env6 | 0.875 | <0.001 | No |
| Com.Env7 | 0.866 | <0.001 | No |
| Com.Env8 | 0.850 | <0.001 | No |
| Com.Env9 | 0.891 | <0.001 | No |
| Com.Env10 | 0.882 | <0.001 | No |
| Com.Env11 | 0.891 | <0.001 | No |
| Com.Env12 | 0.894 | <0.001 | No |
| Com.Env13 | 0.904 | <0.001 | No |
| Com.Env14 | 0.912 | <0.001 | No |
| Com.Env15 | 0.853 | <0.001 | No |
| Com.Env16 | 0.832 | <0.001 | No |
| Com.Env17 | 0.849 | <0.001 | No |

W = Shapiro–Wilk statistic. All items significantly deviated from normality (P < 0.001).

3.4.2 EFA

An exploratory factor analysis was conducted to delineate the latent structure of the 17 Community Environment Function items (Table S7 & Table S8).

**Table S7. Factor Loadings of the Community Environment Function Items**

| **Variable** | **MR1** | **MR4** | **MR3** | **MR5** | **MR2** |
| --- | --- | --- | --- | --- | --- |
| Com.Env1 |  |  | 0.665 |  |  |
| Com.Env2 |  |  | 0.862 |  |  |
| Com.Env3 |  |  | 0.584 |  |  |
| Com.Env4 |  |  | 0.829 |  |  |
| Com.Env5 | 0.752 |  |  |  |  |
| Com.Env6 | 0.906 |  |  |  |  |
| Com.Env7 | 0.835 |  |  |  |  |
| Com.Env8 | 0.752 |  |  |  |  |
| Com.Env9 | 0.561 |  |  |  |  |
| Com.Env10 |  |  |  | 0.720 |  |
| Com.Env11 |  |  |  | 0.776 |  |
| Com.Env12 |  |  |  |  | 0.997 |
| Com.Env13 |  |  |  |  |  |
| Com.Env14 |  |  |  |  | 0.510 |
| Com.Env15 |  | 0.796 |  |  |  |
| Com.Env16 |  | 0.961 |  |  |  |
| Com.Env17 |  | 0.930 |  |  |  |

Factors were extracted using the minimum-residual method and oblimin rotation. Loadings < 0.40 were suppressed. CE1–CE5 denote the five latent community environment factors.

**Table S8. Factor Summary Statistics**

| **Factor** | **SS Loadings** | **Proportion Var** | **Cumulative Var** |
| --- | --- | --- | --- |
| MR1 | 3.080 | 0.181 | 0.181 |
| MR4 | 2.556 | 0.150 | 0.332 |
| MR3 | 2.251 | 0.132 | 0.464 |
| MR5 | 1.381 | 0.081 | 0.545 |
| MR2 | 1.265 | 0.074 | 0.620 |

Note: SS Loadings = sum of squared loadings. Proportion Var = proportion of variance explained. Cumulative Var = cumulative variance explained.

3.4.4 SEM

3.4.4.2 Modification

Table S9 presents the modification indices (MI) and corresponding parameter change statistics for the structural equation model.

**Table S9. Model Relationships and Statistics**

| **lhs** | **op** | **rhs** | **mi** | **epc** | **sepc.lv** | **sepc.all** | **sepc.nox** |
| --- | --- | --- | --- | --- | --- | --- | --- |
| Com.Env5 | ~~ | Com.Env6 | 461.289 | -11.066 | -11.066 | -11.066 | -11.066 |
| CE3 | ~ | Residency | 189.769 | -0.384 | -0.384 | -0.243 | -0.384 |
| CE3 | =~ | Family.function | 141.534 | -0.140 | -0.140 | -0.138 | -0.138 |
| CE5 | =~ | Sleep.quality | 141.172 | -0.436 | -0.436 | -0.418 | -0.418 |
| CE1 | =~ | Sleep.quality | 140.163 | -0.388 | -0.388 | -0.372 | -0.372 |
| Family  function | ~ | CE3 | 131.127 | -0.134 | -0.132 | -0.132 | -0.132 |
| CE4 | =~ | Sleep.quaity | 121.772 | -0.411 | -0.411 | -0.394 | -0.394 |
| CE1 | ~ | Family.  economics | 120.577 | 0.227 | 0.227 | 0.156 | 0.227 |
| Sleep  quality | ~ | Family.function | 120.44 | -0.550 | -0.536 | -0.536 | -0.536 |
| CE3 | =~ | Sleep.quality | 119.178 | -0.393 | -0.393 | -0.376 | -0.376 |

3.4.4.3 Measurement model

Table S10 presents the measurement model estimates, which define the relationships between the latent variables and their observed indicators.

**Table S10. Model Estimates and Statistics**

| **factor** | **item** | **est** | **se** | ***z*** | ***p*** | **std** | **ci.lower** | **ci.upper** |
| --- | --- | --- | --- | --- | --- | --- | --- | --- |
| CE1 | Com.Env6 | 10.302 | 3.647 | 2.825 | 0.005 | 0.995 | 3.155 | 17.449 |
| CE1 | Com.Env5 | 4.401 | 0.315 | 13.957 | 0.000 | 0.975 | 3.783 | 5.019 |
| CE1 | Com.Env7 | 1.708 | 0.058 | 29.665 | 0.000 | 0.863 | 1.595 | 1.821 |
| CE1 | Com.Env8 | 1.698 | 0.057 | 29.62 | 0.000 | 0.862 | 1.586 | 1.811 |
| CE1 | Com.Env9 | 1.246 | 0.043 | 28.851 | 0.000 | 0.78 | 1.161 | 1.331 |
| CE3 | Com.Env4 | 1.879 | 0.089 | 21.148 | 0.000 | 0.883 | 1.705 | 2.054 |
| CE3 | Com.Env2 | 1.803 | 0.081 | 22.238 | 0.000 | 0.874 | 1.644 | 1.962 |
| CE3 | Com.Env1 | 1.71 | 0.084 | 20.434 | 0.000 | 0.863 | 1.546 | 1.874 |
| CE3 | Com.Env3 | 1.186 | 0.052 | 22.629 | 0.000 | 0.765 | 1.084 | 1.289 |
| CE4 | Com.Env15 | 5.13 | 0.817 | 6.275 | 0.000 | 0.982 | 3.528 | 6.732 |
| CE4 | Com.Env16 | 2.459 | 0.13 | 18.859 | 0.000 | 0.926 | 2.203 | 2.714 |
| CE5 | Com.Env10 | 2.209 | 5.094 | 0.434 | 0.664 | 0.911 | -7.774 | 12.193 |
| SRH | SF.Total | 1.000 | 0.000 | nan | nan | 1.000 | 1.000 | 1.000 |
| Sleep  quality | Sleep.  quality | 1.000 | 0.000 | nan | nan | 1.000 | 1.000 | 1.000 |
| Family  function | family.  function | 1.000 | 0.000 | nan | nan | 1.000 | 1.000 | 1.000 |
